# Effects of smoking cessation on individuals with COPD: a systematic review and meta-analysis

**DOI:** 10.3389/fpubh.2024.1433269

**Published:** 2024-12-11

**Authors:** Zihan Wang, Yifan Qiu, Xiang Ji, Liang Dong

**Affiliations:** ^1^Department of Respiratory, Shandong Qianfoshan Hospital, Cheeloo College of Medicine, Shandong University, Jinan, China; ^2^Department of Respiratory and Critical Care Medicine, Research Center for Chronic Airway Diseases, Peking University Health Science Center, Peking University Third Hospital, Beijing, China; ^3^Department of Respiratory, Shandong Provincial Qianfoshan Hospital, The First Affiliated Hospital of Shandong First Medical University, Shandong Institute of Respiratory Diseases, Shandong University, Jinan, China

**Keywords:** smoking cessation, COPD, pulmonary function, mMRC scale, partial oxygen pressure, 6-MWT, mortality

## Abstract

**Objective:**

Despite smoking being a significant risk factor in the occurrence and progression of chronic obstructive pulmonary disease (COPD), no comprehensive analysis has been conducted to determine the potential benefits of smoking cessation for patients with established COPD or identify specific indicators that may be improved. The aim of our meta-analysis was to elucidate the positive impact of smoking cessation on COPD.

**Methods:**

We conducted a comprehensive search of PubMed, EMBASE, Web of Science, Cochrane Library, CNKI, Wan Fang and VIP databases to identify studies that met our eligibility criteria from inception up to 1, May 2024. Data were extracted independently by two authors and pooled using a random-effects model. Study quality was evaluated using the Newcastle-Ottawa Scale (NOS).

**Results:**

Preliminary screening of publications gave a total of 13,460 documents after which the repetitive and non-compliant studies were removed. Eventually, 11 studies were included for follow-up analysis. The pooled results showed that cessation of smoking produced significant improvements in forced expiratory volume in one second (FEV1)% predicted (MD = 6.72, 95% CI, 4.55–8.89, *P* < 0.001; I^2^ = 53%), FEV1/forced vital capacity (FVC) (MD = 6.82, 95% CI, 5.09-8.54, *P* < 0.001; I^2^ = 0%), modified Medical Research Council (mMRC) (MD = −0.49, 95% CI, −0.95–−0.02, *P* = 0.040; I^2^ = 73%), 6-minute walk test (6-MWT) (MD = 64.46, 95% CI 14.60-114.32, *P* = 0.010; I^2^ = 94%), partial oxygen pressure (MD = 1.96, 95% CI, 1.03-2.89, *P* < 0.001; I^2^ = 0%), mortality (RR = 0.75, 95% CI, 0.56-1.00, *P* = 0.05; I^2^ = 44%).

**Conclusion:**

Our meta-analysis presented suggestive evidence that smoking cessation offered significant benefits to COPD patients, notably in the improvement of specific key indicators of pulmonary function (FEV1% predicted, FEV1/FVC), alleviating symptoms, enhancing exercise tolerance, and could reduce mortality.

**PROSPERO registration:**

https://www.crd.york.ac.uk/prospero/, identifier: CRD42022384123.

## 1 Introduction

Chronic obstructive pulmonary disease (COPD) is a non-communicable respiratory illness with high global incidence and mortality rates (1, 2). It has been estimated that over 3 million people worldwide die from COPD annually, resulting in significant societal and health-care burdens (3). Numerous risk factors may contribute to the onset and progression of COPD, including environmental exposures (such as tobacco smoking, biomass smoke and indoor air pollutants) (4), aging (5), and genetic predispositions (6), among others (7). However, tobacco use as a risk factor remains an actionable intervention. The Global Initiative for Chronic Obstructive Lung Disease (also known as GOLD) committee proposed quitting smoking as a necessary and evidence-based step for the prevention and treatment of COPD in 2001 (8). While the underlying molecular mechanism of smoking-related COPD is only partially understood, the exact quantitative factors associated with smoking that affect the prognosis of patients with COPD remain unknown. The previous study indicated that quitting smoking can delay the natural progression of COPD (9), with better results achieved when the cessation of smoking occurs at an early age (10). Furthermore, studies have demonstrated that quitting smoking can significantly alleviate respiratory symptoms in the short term and improve lung function, quality of life, and survival rate in the long term among patients with COPD(9, 11). However, several studies have suggested that there is persistent lung inflammation and a decline in lung function observed in mice following smoking cessation interventions (12, 13).

Above all, there exists a dearth of comprehensive systematic reviews that substantiate the tangible advantages of smoking cessation for COPD patients. In light of this, our research aims to conduct a comprehensive systematic review and meta-analysis that elucidates the multifaceted impact of smoking cessation on COPD.

## 2 Methods

Before conducting this meta-analysis, we registered our study on PROSPERO (https://www.crd.york.ac.uk/PROSPERO/) with the registration code CRD42022384123. The Preferred Reporting Items for Systematic Reviews and Meta-Analyses (PRISMA) guidelines were strictly adhered to when reporting our findings (Supplementary material 1).

### 2.1 Search strategy

We searched for publications in PubMed, EMBASE, Web of Science, Cochrane Library, CNKI, Wan Fang, and VIP databases from their inception up to 1, May 2024. The detailed search strategy is presented in Supplementary material 2. Two investigators (W.Z.H, Q.Y.F) independently searched and screened all relevant articles, including systematic review studies, cohort studies, books, documents, etc. We evaluated the relevance of the full text of the articles and conducted independent screening according to the following criteria.

### 2.2 Inclusion and exclusion criteria

Studies were included if they matched the following criteria: (1) Studies related to patients with COPD and smoking cessation; (2) The research type of the study included is a prospective or retrospective study; (3) The individuals in the included studies were required to be satisfied that they had spirometrically confirmed COPD enrolled in the study (FEV1/FVC < 0.7 post-bronchodilation) or they had physician-diagnosed COPD before. (4) The under-study individuals groups quit smoking, while the control groups continued to smoke; (5) The observation time of the participants was more than 3 months; (6) The included research confirmed whether they were in smoking status through biological measures such as exhaled CO level or self-report; (7) The study was published in English or Chinese.

The exclusion criteria include: (1) The research types were *in vitro* studies or animal studies, reviews, case reports, and meta-analyses. (2) The full text could not be obtained or the information used to calculate the results was insufficient; (3) The research data provided by the articles was not complete; (4) Repetitive articles; (5) The control group (continuous-smoking group) had ever tried to quit smoking.

### 2.3 Data extraction and quality assessment

Two authors extracted data independently and screened eligible articles according to the title, keyword, abstract, and full text of the studies. Subsequently, the articles which met the standards were included. The data extracted and analyzed included the author of the article, sample size, research types, intervention protocol, and outcome indicators of the subjects. When authors have differing opinions on data extraction, they should fully discuss the problem of disagreement and even seek the help of a third person to make a judgment. The studies with incomplete data were excluded.

The quality of the included studies was evaluated using the NOS scale by two independent reviewers (W.Z.H and Q.Y.F). The scale in question encompasses a trifold assessment of selection, comparability and outcomes, each of which comprises several dimensions. Selection, for instance, is evaluated based on four criteria, including the representativeness of the cohort, selection of the unexposed cohort (if applicable), exposure verification, and demonstration that the outcome of interest was absent at baseline. Comparability, on the other hand, takes into account the similarities between the cohorts for study design and analysis. Finally, the results dimension scrutinizes the assessment of the results, the follow-up period, and the sufficiency of the follow-up of the cohorts. The NOS scale uses a “star” rating system (ranging from 0 stars to 9 stars) to judge quality: 7–9 stars denote “high” quality, 4–6 stars denote “medium” quality and 0–3 stars represent “low” quality (14).

### 2.4 Statistics analysis

RevMan 5.3 (www.cochrane.org/) and STATA 15.1 (StataCorp, Cary, NC, USA) were employed for data analyses in this meta-analysis study. For dichotomous data, we estimated the summary risk ratio (RR) with a 95% confidence interval (95% CI), and for continuous data, we estimated the mean difference (MD) with a 95% CI. The pooled effect sizes (ESs) were determined using a randomized-effect model and shown in a forest plot. The I^2^ statistic and Q-Cochran test were used to examine heterogeneity across studies. Cochran's Q test *P* < 0.1 was considered significant to assume apparent heterogeneity. The I^2^ < 25%, 25% < I^2^ < 50%, and I^2^ > 50% were considered as low heterogeneity, moderate heterogeneity, and high heterogeneity, respectively. Subgroup analysis was based on the duration of cessation and mean age of initial cessation for COPD patients. Had we included more than 10 studies in any outcome, we would have created a funnel plot to screen for publication bias. A sensitivity analysis was conducted to assess the stability of the results by excluding one study each time. Statistical significance was set at *P* < 0.05.

## 3 Results

### 3.1 Search results

**Figure 1:**
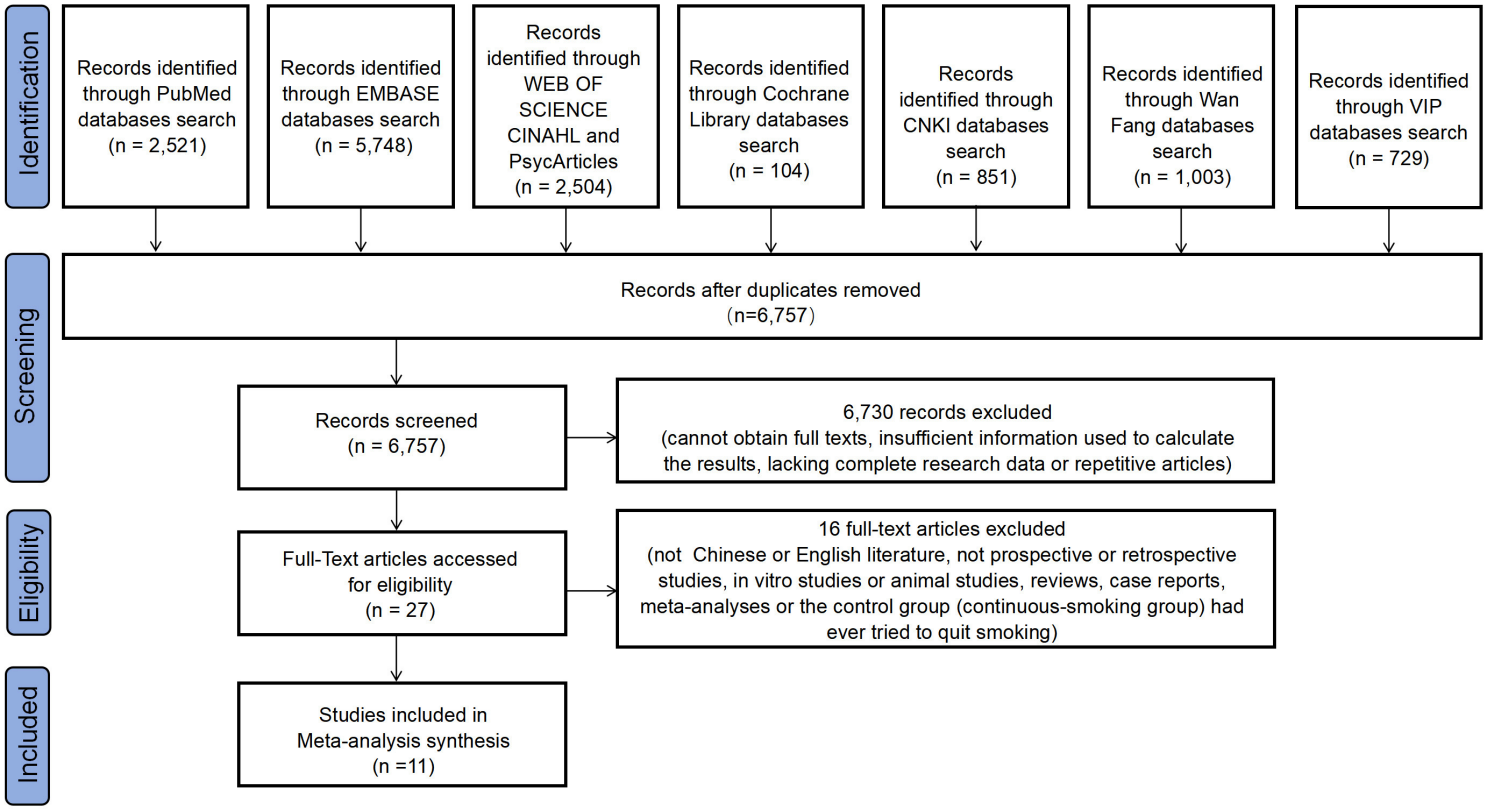
Flow diagram of study selection.

Initially, a total of 13, 460 records were retrieved from the database. After removing duplicates (n = 6,703), 6,757 studies were reviewed and 27 studies remained. Subsequently, sixteen studies were excluded based on the reasons shown in the flowchart. Finally, 11 studies were selected for inclusion in this meta-analysis. The PRISMA flowchart of the literature search and related screening process of the meta-analysis study is presented below (Figure 1).

### 3.2 Study characteristics

Patient and study characteristics of the 11 included studies are described in Table 1. Each of the included studies compared quitters and non-quitters for more than 3 months. Among these studies, eight conducted pulmonary function tests, while three employed dyspnea assessing indicators, including the mMRC scale (15) and 6-MWT (16). Furthermore, two studies recorded the measurement of partial pressure of oxygen in blood gas analysis, and four studies presented statistics of mortality. The NOS quality score showed that the included studies had a high-quality rating (Table 2).

**Table 1:** The characteristics of included studies.

**References**	**Study design**	**Country**	**Sample size**		**Age (mean years)**		**Baseline FEV1% predicted (mean%)**		**Time duration**	**Biological measures for smoking status**	**Outcome index**
			**I**	**C**	**I**	**C**	**I**	**C**			
Anthonisen et al. (24)	Prospective study	America/Canada	3,923	1,964	48.5	48.4	75.1	75.1	5 years	Exhaled CO level	⑤
Bai et al.(17)	Retrospective study	China	92	112	67.4	68.4	44.5	42.6	5 years	Self-report	①④⑤
Doo et al.(25)	Prospective study	South Korea	530	1,210	66.7	63.8	NA	NA	12 years	Self-report	⑤
Lei et al. (9)	Prospective study	China	51	51	61.1	61.5	50.8	51.3	10 years	Self-report	①⑤
Liu et al. (18)	Prospective study	China	30	30	74.3	73.5	35.8	34.6	3 months	Self-report	①②③
Liu et al. (21)	Prospective study	China	44	44	52.6	52.1	52.4	51.9	1 year	Self-report	①②
Le Mao et al. (23)	Prospective study	France	42	39	57.6	56.0	NA	NA	52 weeks	Exhaled CO level	①②③
Pezzuto et al. (11)	Retrospective study	Italy	120	80	41.5	42.0	75.2	77.3	3 months	Exhaled CO level	①②④
Saetia et al. (22)	Prospective study	Thailand	30	30	Female-67.6 Male-63.77	Female-67.4 Male-62.58	NA	NA	11 months	Self-report	①③
Zhang et al. (20)	Prospective study	China	166	67	60.5	60.5	53.9	54.1	1 year	Exhaled CO level	①②
Zhou et al. (19)	Prospective study	China	60	40	59.3	63.5	60.2	57.9	6 months	Self-report	①

C, Control group, i.e., continuous smoking group; I, Intervention group, I.e., smoking cessation group.

①Pulmonary function ②mMRC ③6-MWT ④Partial oxygen pressure (mm Hg) ⑤Mortality.

**Table 2:** Quality assessment of included studies by NOS.

**References**	**Selection**				**Comparability**		**Outcome**			**Stars**
	**Q1**	**Q2**	**Q3**	**Q4**	**Q5**	**Q6**	**Q7**	**Q8**	**Q9**	
Anthonisen et al. (24)	^*^	^*^	^*^	^*^	^*^	^*^	^*^	^*^	^*^	9
Bai et al. (17)	^*^	^*^	-	^*^	^*^	^*^	-	^*^	^*^	7
Doo et al. (25)	^*^	^*^	-	^*^	^*^	^*^	-	^*^	^*^	7
Lei et al. (9)	^*^	^*^	-	^*^	^*^	^*^	-	^*^	^*^	7
Liu et al. (18)	^*^	^*^	-	^*^	^*^	^*^	-	^*^	^*^	7
Liu et al. (21)	^*^	^*^	-	^*^	^*^	^*^	-	^*^	^*^	7
Le Mao et al. (23)	^*^	^*^	^*^	^*^	^*^	^*^	^*^	^*^	^*^	9
Pezzuto et al. (11)	^*^	^*^	^*^	^*^	^*^	^*^	^*^	^*^	^*^	9
Saetia et al. (22)	^*^	^*^	-	^*^	^*^	^*^	-	^*^	^*^	7
Zhang et al. (20)	^*^	^*^	^*^	^*^	^*^	^*^	^*^	^*^	^*^	9
Zhou et al. (19)	^*^	^*^	-	^*^	^*^	^*^	-	^*^	^*^	7

### 3.3 Synthesized results

#### 3.3.1 Effect of smoking cessation on pulmonary function in patients with COPD

##### 3.3.1.1 FEV1% predicted

**Figure 2:**
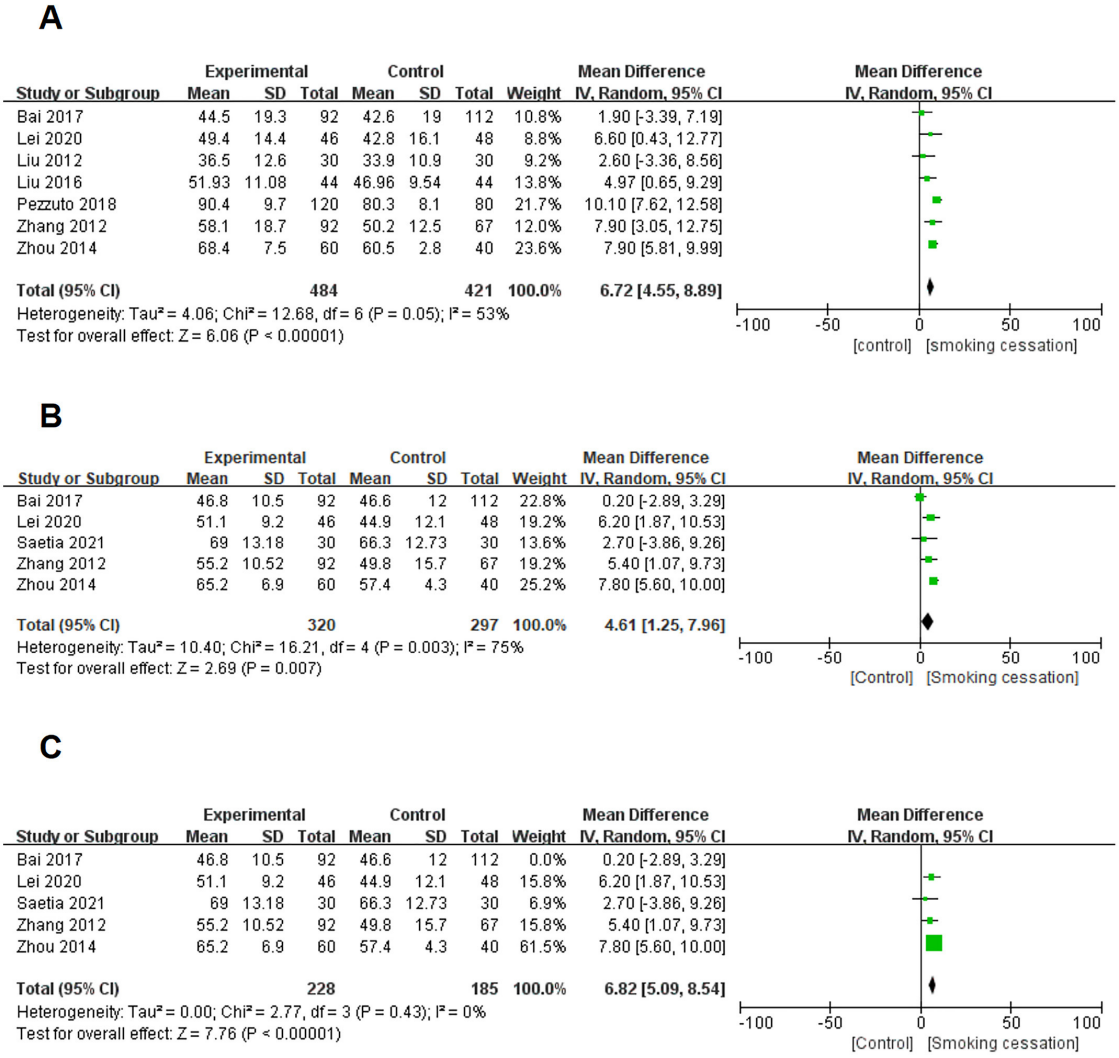
Forest plots for effects of smoking cessation on lung function for individuals with COPD. **(A)** Effects of smoking cessation on FEV1% predicted. **(B)** Effects of smoking cessation on FEV1/FVC were assessed before excluding studies with significant heterogeneity. **(C)** Effects of smoking cessation on FEV1/FVC were assessed after excluding studies with significant heterogeneity.

Seven studies (9, 11, 17–21) were pooled for meta-analysis of FEV1% predicted in patients with COPD. Compared with COPD patients who continued smoking, FEV1% predicted of COPD patients who quit smoking increased significantly (MD = 6.72, 95% CI, 4.55–8.89, *P* < 0.001) (Figure 2A), the between-study heterogeneity of which was I^2^ = 53% (*P* = 0.050).

##### 3.3.1.2 FEV1/FVC

Five eligible studies (9, 17, 19, 20, 22) were included to expound the impact of smoking cessation on FEV1/FVC in the lung function of COPD patients. The result revealed that the between-study heterogeneity was substantial (I^2^ = 75%, *P* = 0.003; Figure 2B). The above results showed that the heterogeneity of the research results was high. Therefore, by removing the studies one by one and conducting the analysis, we finally determined that the study, Bai 2017, was the main source of heterogeneity in this analysis. Next, the above data result was removed, and the remaining four studies were re-analyzed based on FEV1/FVC. Compared with COPD patients who continued smoking, FEV1/FVC of COPD patients who quit smoking increased significantly (MD = 6.82, 95% CI, 5.09–8.54, *P* < 0.001; I^2^ = 0%) (Figure 2C).

#### 3.3.2 Effect of smoking cessation on dyspnea assessing indicators in patients with COPD

##### 3.3.2.1 mMRC scale

**Figure 3:**
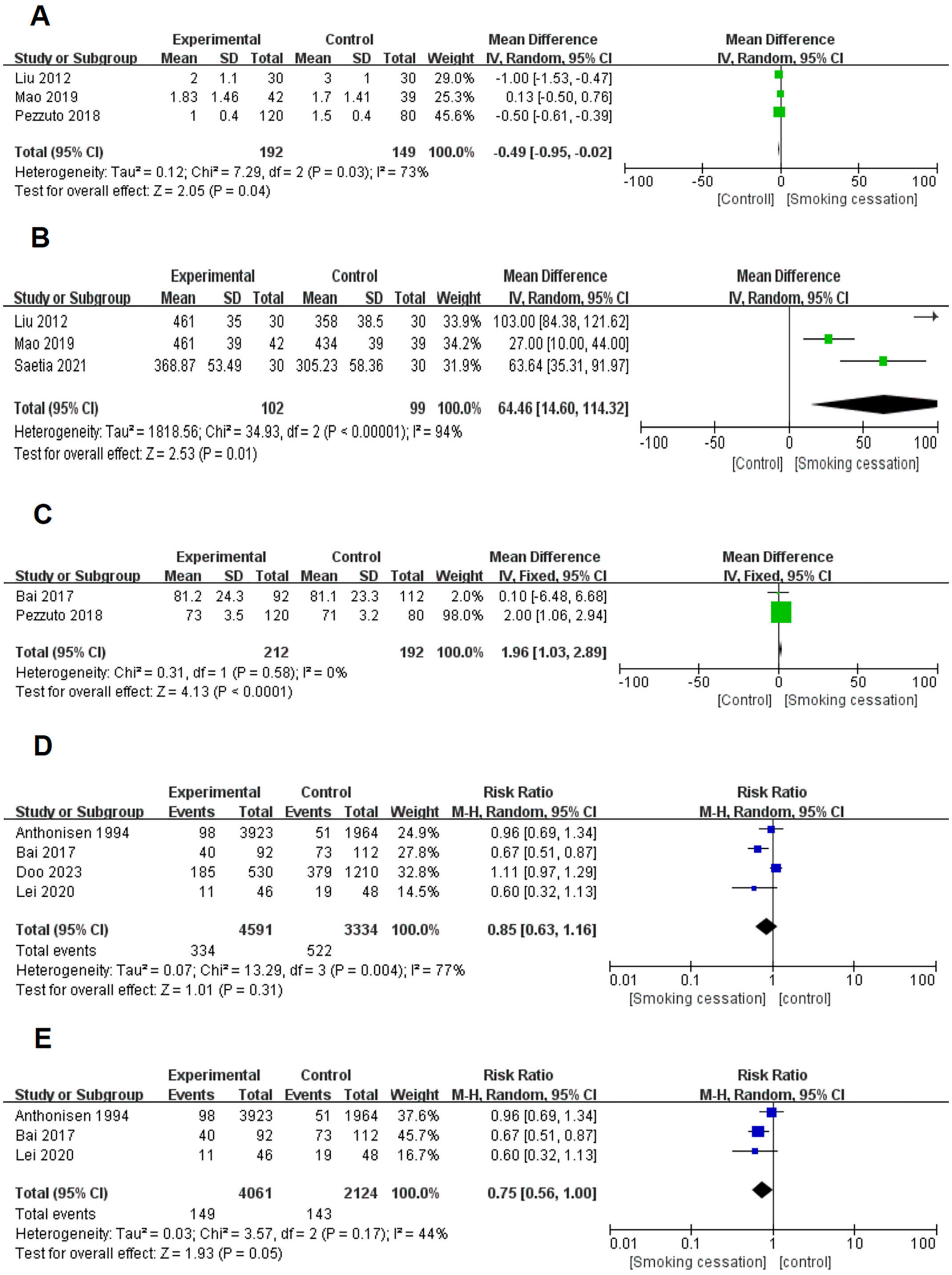
Forest plots for effects of smoking cessation on several objective evaluation index for individuals with COPD. **(A)** Effects of smoking cessation on mMRC. **(B)** Effects of smoking cessation on 6-MWT. **(C)** Effects of smoking cessation on partial oxygen pressure (mm Hg). **(D)** Effects of smoking cessation before excluding studies with significant heterogeneity. **(E)** Effects of smoking cessation on mortality were assessed after excluding studies with significant heterogeneity.

Three studies (11, 18, 23) were included to conduct the meta-analysis of the impact of smoking cessation on the mMRC scale of COPD patients. The result revealed that the between-study heterogeneity was substantial (I^2^ = 73%, *P* = 0.030; Figure 3A). However, despite conducting sensitivity analysis, none of the studies exerted any influence on the heterogeneity observed in the final results. Thus, the analysis of the 3 existing studies showed that smoking cessation had an impact on the mMRC score of COPD patients (MD = −0.49, 95% CI, −0.95–−0.02, *P* = 0.040) (Figure 3A).

##### 3.3.2.2 Six-minute walking test

Three studie s(18, 22, 23) were combined for the meta-analysis of 6-MWT in patients with COPD. Patients with COPD who quit smoking showed significantly better 6-MWT outcomes than those who continued to smoke. (MD = 64.46, 95% CI 14.60–114.32, *P* = 0.010; I^2^ = 94%, *P* < 0.001. Figure 3B). Due to the large heterogeneity, we conducted a sensitivity analysis of the one-by-one exclusion method, but there was no eligible study for removal.

#### 3.3.3 Effect of smoking cessation on blood gas analysis in patients with COPD

Two studies (11, 17) pooled results for meta-analysis of blood gas analysis in patients with COPD and showed that the partial oxygen pressure (mm Hg) of COPD patients who quit smoking was significantly higher than that of COPD patients who continued smoking (MD = 1.96, 95% CI, 1.03–2.89, *P* < 0.001; I^2^ = 0%, *P* = 0.580) (Figure 3C).

#### 3.3.4 Effect of smoking cessation on mortality of patients with COPD

Based on the preliminary meta-analysis of the four studies (9, 17, 24, 25), it was found that there was a significant heterogeneity in the results (I^2^ = 77%, *P* = 0.004) (Figure 3D). As a result, a sensitivity analysis was performed, which revealed that the Doo et al. study was the main source of heterogeneity in the results. To address this, the Doo et al. study was excluded and a meta-analysis was conducted on the remaining three studies. The analysis indicated that smoking cessation could have an impact on reducing all-cause mortality in patients with COPD (RR = 0.75, 95% CI, 0.56–1.00, *P* = 0.050; I^2^ = 44%, *P* = 0.170) (Figure 3E).

## 4 Subgroup analysis for the outcomes

To further explore the effects of duration of smoking cessation (<1 year/≥1 year) and mean age of initiation (<60-year-old/≥60-year-old) on patients with COPD, we conducted a subgroup analysis of some of the results that could be analyzed by using this method (Supplementary Figures 1A–D, 2A–D). It is noteworthy that patients with COPD who ceased smoking for less than a year manifested more significant improvements in 6-MWT compared with those who quit for over a year. Furthermore, COPD patients who quit smoking at an average age of <60 years displayed better FEV1% than those who commenced the quitting process at an average age of over 60 years. Howerver, the outcomes were inverted for 6-MWT patients.

## 5 Discussion

To the best of our knowledge, this is the first systematic review to investigate the impact of smoking cessation on a specific population of patients diagnosed with COPD. COPD remains a pervasive respiratory disorder that presents an enduring challenge in the 21st century, given the aging population and escalating environmental pollution. Besides, COPD frequently coexists with a range of comorbid conditions, thereby exacerbating the overall medical burden (26). While it is widely acknowledged that cigarette smoking plays a pivotal role in the onset and progression of this condition, there persists a higher prevalence of smoking among COPD patients (27, 28), highlighting the lack of awareness regarding the importance of smoking cessation or excessive tobacco dependence for those already afflicted. Our study conducted a comprehensive systematic review and meta-analysis of 11 studies to assess the impact of smoking cessation on patients with COPD, emphasizing the imperative nature of smoking cessation for individuals with COPD. The findings indicate that smoking cessation can enhance the pulmonary function of COPD patients, particularly in terms of FEV1% predicted and FEV1/FVC. Additionally, it can improve patients' endurance during physical exercise and increase the partial pressure of blood gas analysis for oxygen. From our findings, we also conclude that quitting smoking may reduce the mortality due to COPD.

The characteristic manifestations of COPD are progressive airway inflammation and incurable remodeling, primarily induced by cigarette smoke exposure (27). Chronic inhalation of cigarette smoke can activate pattern recognition receptors, such as Toll-like receptors, resulting in an innate immune response characterized by increased numbers of neutrophils and macrophages in the lungs, activation of airway epithelial cells, and mucus secretion (27). Additionally, cigarette smoke can induce excessive release of reactive oxygen species (ROS) from neutrophils and macrophages (29), thereby impairing antiprotease mechanisms and accelerating pulmonary tissue degradation, consequently facilitating the progression of emphysema (30).

Fortunately, numerous studies have demonstrated that smoking cessation not only alleviates lung dysfunction and inflammation but also delays the decline of lung function in individuals with sustained lung function injury due to COPD (31–33). In our study, we used FEV1% predicted and FEV1/FVC as indicators for evaluating lung function. Specifically, we analyzed seven studies that utilized the percentage of predicted FEV1 as an evaluation criterion and five studies that employed the FEV1/FVC. The synthesized outcomes were consistent with prior research, indicating that quitting smoking can ameliorate the pulmonary function status in individuals with COPD. During the progress of the meta-analysis, it was found that Bai et al.'s retrospective study exhibited a significant impact on the heterogeneity of the analysis outcomes, leading to its exclusion from the analysis. This disparity may be attributed to the study's retrospective nature as opposed to the prospective designs of the other four studies under scrutiny.

The assessment of breathlessness in patients with COPD is typically performed using the mMRC scale, a questionnaire that consists of five grades (34). Previous research had demonstrated that compared to continuous smokers, individuals with COPD who quit smoking had a higher likelihood of experiencing improvements in dyspnea, relief from cough symptoms, and an increase in oxygenation index. Conversely, persistent smoking results in continuous stimulation of cigarette smoke that leads to progressive exacerbation of airway inflammation and irreversible remodeling of the airway. This ultimately causes a worsening of dyspnea symptoms over time. Our meta-analysis, comprising four studies, indicates that smoking cessation positively impacts the alleviation of respiratory discomfort symptoms, particularly breathlessness, in patients with COPD. Due to the high heterogeneity of the results which cannot be eliminated, additional available studies will be included to analyze the effect of smoking cessation on improving dyspnea symptoms in patients with COPD. Additionally, the 6-MWT served as a valuable qualitative tool for assessing exercise tolerance and monitoring treatment effectiveness and disease progression in patients with COPD. Our meta-analysis of three studies revealed that smoking cessation significantly improved the 6-MWT values among COPD patients, suggesting its efficacy in enhancing exercise tolerance. Additionally, sufficient oxygen supply and efficient diffusion into tissues were indispensable for survival. The assessment of lung ventilation efficiency commonly relies on blood gas analysis, with oxygen partial pressure serving as a crucial indicator. Our meta-analysis of three cohort studies established that smoking cessation played a pivotal role in enhancing blood gas levels among patients with COPD(35). The increase in blood oxygen supply reduced the stimulation of peripheral chemoreceptors in the carotid body. As a result, the overactivation of the respiratory center was alleviated, leading to a more stable breathing pattern and improved respiratory efficiency, which further elucidated how quitting smoking could alleviate respiratory symptoms associated with COPD.

The progressive annual escalation in the mortality rate among patients with COPD is a matter of concern. Our findings indicate that quitting smoking can lead to a decrease in overall mortality among individuals with COPD, although the statistical significance is borderline, this result holds substantial clinical importance. This outcome could be attributed to the limited sample sizes in each study and the overall number of studies that were included.

Additionally, our subgroup analysis based on cessation duration and age of initial cessation revealed variations in FEV1/FVC% or 6-MWT among the groups. However, these results exhibited inconsistencies with each other, potentially attributed to the limited number of studies included in each subgroup, thereby compromising the reliability of the findings.

Our current study had several limitations. Firstly, the meta-analysis results did not reveal significant differences between male and female adults in terms of quitting smoking, which raises uncertainty about whether gender differences could impact COPD treatment outcomes. Secondly, the limited number of studies included may have resulted in publication bias and the small sample sizes in some reference studies may have led to slight deviations in outcomes. Thirdly, due to variations in intervention and observation times across studies, there was inevitable heterogeneity.

## 6 Conclusion

In conclusion, our meta-analysis provides compelling evidence to support the recommendation of smoking cessation as a crucial strategy in accordance with the GOLD guidelines, demonstrating significant benefits for patients with COPD. The comparative analysis between individuals who ceased smoking and those who continued revealed significant enhancements, including specific key indicators of pulmonary function (FEV1% predicted, FEV1/FVC), and exercise capacity among patients with COPD. Besides, smoking cessation also could reduce the mortality of COPD patients. Therefore, early smoking cessation among individuals diagnosed with COPD was strongly advocated to enhance their overall quality of life.

## Data Availability

The raw data supporting the conclusions of this article will be made available by the authors, without undue reservation.
