# Supplementary material for: Effects of smoking cessation on individuals with COPD: a systematic review and meta-analysis
Source: Front Public Health. 2024 Dec 11;12:1433269. doi: 10.3389/fpubh.2024.1433269 (PMC11668769; doi:10.3389/fpubh.2024.1433269)
Supplement: Supplementary file 2 [file Table_2.docx]

**Search strategy of each online database**

| **Online databases** | **Search number** | **Search Strategy** |
| --- | --- | --- |
| Pubmed | #1 | **((((((((((Pulmonary Disease, Chronic Obstructive[MeSH Terms]) OR (Chronic Obstructive Lung Disease)) OR (Chronic Obstructive Pulmonary Diseases)) OR (Chronic Obstructive Pulmonary Diseases)) OR (COPD)) OR (Chronic Obstructive Airway Disease)) OR (Chronic Obstructive Airway Disease)) OR (Airflow Obstruction, Chronic)) OR (Airflow Obstructions, Chronic)) OR (Chronic Airflow Obstructions)) OR (Chronic Airflow Obstruction)** |
|  | #2 | **((((((((((Smoking Cessation[MeSH Terms]) OR (Cessation, Smoking)) OR (Smoking Cessations)) OR (Stopping Smoking)) OR (Smoking, Stopping)) OR (Giving Up Smoking)) OR (Smoking, Giving Up)) OR (Smoking, Giving Up)) OR (Up Smoking, Giving)) OR (Up Smoking, Giving)) OR (Up Smoking, Giving)** |
|  | #3 | #1 AND #2 |
| Embase | #1 | 'chronic obstructive lung disease'/exp |
|  | #2 | 'chronic airflow obstruction':ab,ti OR 'chronic airway obstruction':ab,ti OR 'chronic obstructive bronchopulmonary disease':ab,ti OR 'chronic obstructive lung disorder':ab,ti OR 'chronic obstructive pulmonary disease':ab,ti OR 'chronic obstructive pulmonary disorder':ab,ti OR 'chronic obstructive respiratory disease':ab,ti OR 'chronic pulmonary obstructive disease':ab,ti OR 'chronic pulmonary obstructive disorder':ab,ti OR 'copd':ab,ti OR 'lung chronic obstructive disease':ab,ti OR 'lung disease, chronic obstructive':ab,ti OR 'obstructive chronic lung disease':ab,ti OR 'obstructive chronic pulmonary disease':ab,ti OR 'obstructive lung disease, chronic':ab,ti OR 'pulmonary disease, chronic obstructive':ab,ti OR 'pulmonary disorder, chronic obstructive':ab,ti OR 'chronic obstructive lung disease':ab,ti |
|  | #3 | #1 OR #2 |
|  | #4 | 'smoking cessation'/exp |
|  | #5 | #4 OR #5 |
|  | #6 | 'abstination, smoking':ab,ti OR 'abstinence from nicotine':ab,ti OR 'abstinence from smoking':ab,ti OR 'abstinence from tobacco':ab,ti OR 'cessation, smoking':ab,ti OR 'dehabituation, smoking':ab,ti OR 'nicotine abstination':ab,ti OR 'nicotine abstinence':ab,ti OR 'nicotine cessation':ab,ti OR 'nicotine withdrawal':ab,ti OR 'quit smoking':ab,ti OR 'smoking abstinence':ab,ti OR 'smoking dehabituation':ab,ti OR 'smoking, stopping':ab,ti OR 'stop smoking':ab,ti OR 'stopping smoking':ab,ti OR 'tobacco use cessation':ab,ti OR 'smoking cessation':ab,ti |
|  | #7 | #3 AND #6 |
| Web of science | #1 | (ALL=(chronic obstructive pulmonary disease)) OR TS=('chronic airflow obstruction' OR 'chronic airway obstruction' OR 'chronic obstructive bronchopulmonary disease' OR 'chronic obstructive lung disorder' OR 'chronic obstructive pulmonary disease' OR 'chronic obstructive pulmonary disorder' OR 'chronic obstructive respiratory disease' OR 'chronic pulmonary obstructive disease' OR 'chronic pulmonary obstructive disorder' OR 'copd' OR 'lung chronic obstructive disease' OR 'lung disease, chronic obstructive' OR 'obstructive chronic lung disease' OR 'obstructive chronic pulmonary disease' OR 'obstructive lung disease, chronic' OR 'pulmonary disease, chronic obstructive' OR 'pulmonary disorder, chronic obstructive' OR 'chronic obstructive lung disease') |
|  | #2 | (ALL=(smoking cessation)) OR ALL=('abstination, smoking' OR 'abstinence from nicotine' OR 'abstinence from smoking' OR 'abstinence from tobacco' OR 'cessation, smoking' OR 'dehabituation, smoking' OR 'nicotine abstination' OR 'nicotine abstinence' OR 'nicotine cessation' OR 'nicotine withdrawal' OR 'quit smoking' OR 'smoking abstinence' OR 'smoking dehabituation' OR 'smoking, stopping' OR 'stop smoking' OR 'stopping smoking' OR 'tobacco use cessation' OR 'smoking cessation') |
|  | #3 | #1 AND #2 |
| Cochrane Library | #1 | MeSH descriptor: [Pulmonary Disease, Chronic Obstructive] explode all trees |
|  | #2 | MeSH descriptor: [Smoking cessation] explode all trees |
|  | #3 | #1 AND #2 |
| CNKI (Chinese database) |  | （篇关摘：慢性阻塞性肺疾病（模糊））AND（篇关摘：戒烟（模糊）） |
| Wan Fang (Chinese database) |  | （中英文扩展&主题词扩展）： 全部:(慢性阻塞性肺疾病) and全部:(戒烟) |
| VIP (Chinese database) |  | 任意字段=慢性阻塞性肺疾病（模糊） AND 任意字段=戒烟（模糊） |
