# Supplementary figures and images for: Effects of smoking cessation on individuals with COPD: a systematic review and meta-analysis
Source: Front Public Health. 2024 Dec 11;12:1433269. doi: 10.3389/fpubh.2024.1433269 (PMC11668769; doi:10.3389/fpubh.2024.1433269)

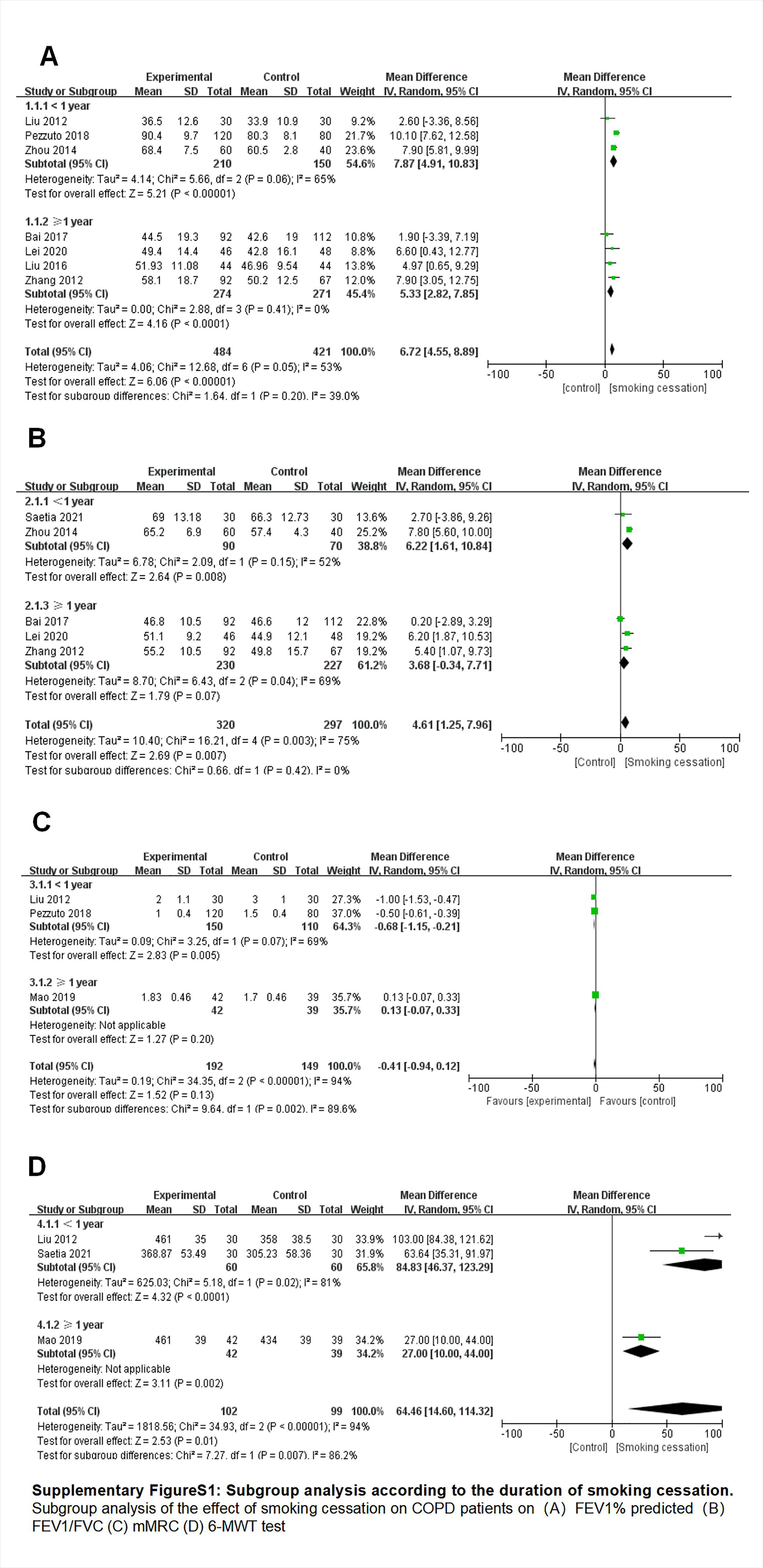

Supplement: Supplementary file 3 [file Image_1.tif]

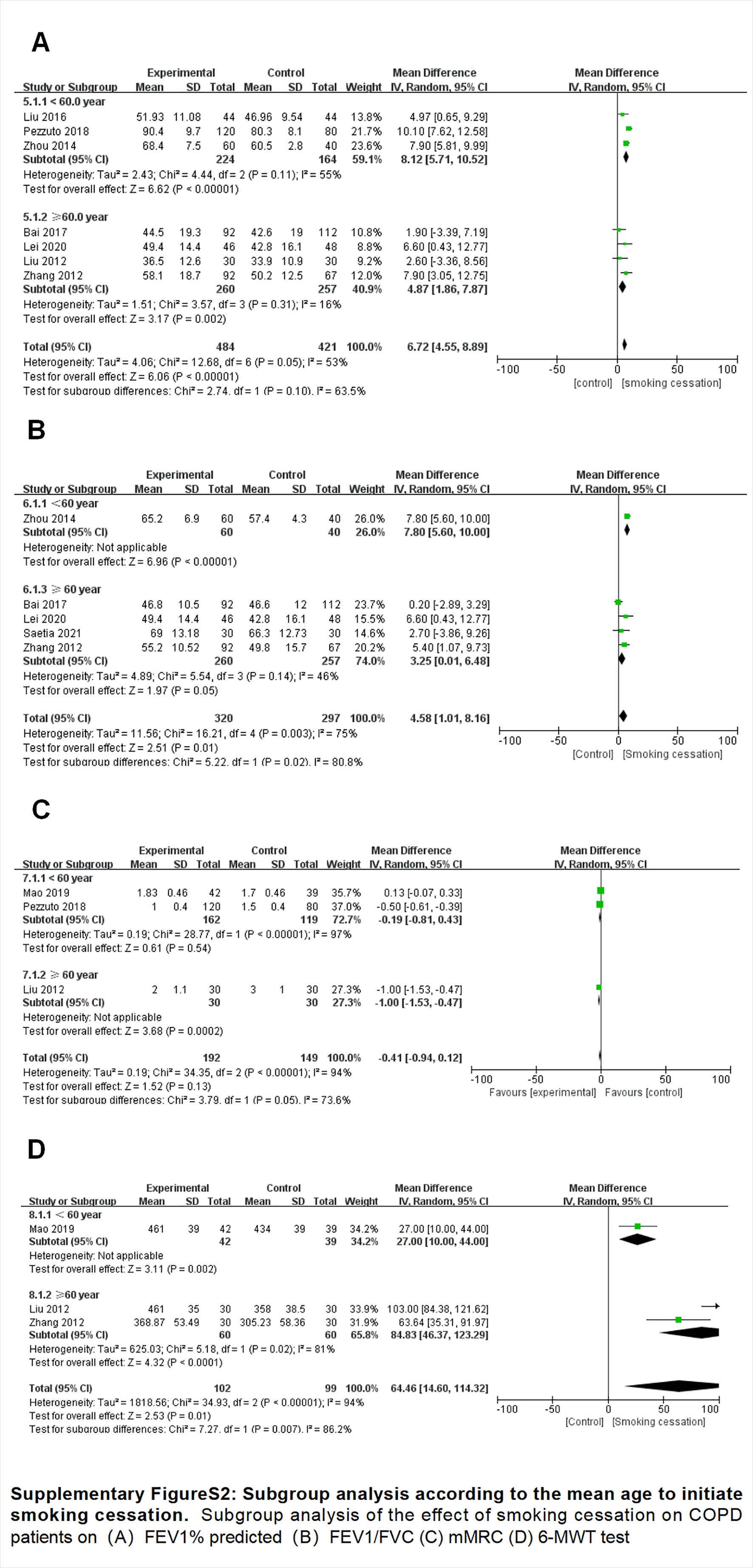

Supplement: Supplementary file 4 [file Image_2.tif]
